# Peripheral Surgical Wounding and Age-Dependent Neuroinflammation in Mice

**DOI:** 10.1371/journal.pone.0096752

**Published:** 2014-05-05

**Authors:** Zhipeng Xu, Yuanlin Dong, Hui Wang, Deborah J. Culley, Edward R. Marcantonio, Gregory Crosby, Rudolph E. Tanzi, Yiying Zhang, Zhongcong Xie

**Affiliations:** 1 Geriatric Anesthesia Research Unit, Department of Anesthesia, Critical Care and Pain Medicine, Massachusetts General Hospital and Harvard Medical School, Charlestown, Massachusetts; 2 Department of Anesthesia, Beijing Chaoyang Hospital, Capital Medical University, Beijing, P. R. China; 3 Department of Anesthesia, Brigham & Women’s Hospital and Harvard Medical School, Boston, Massachusetts; 4 Divisions of General Medicine and Primary Care and Gerontology, Department of Medicine, Beth Israel Deaconess Medical Center and Harvard Medical School, Boston, Massachusetts; 5 Genetics and Aging Research Unit, MassGeneral Institute for Neurodegenerative Disease, Department of Neurology, Massachusetts General Hospital and Harvard Medical School, Charlestown, Massachusetts; University of Virginia, United States of America

## Abstract

Post-operative cognitive dysfunction is associated with morbidity and mortality. However, its neuropathogenesis remains largely to be determined. Neuroinflammation and accumulation of β-amyloid (Aβ) have been reported to contribute to cognitive dysfunction in humans and cognitive impairment in animals. Our recent studies have established a pre-clinical model in mice, and have found that the peripheral surgical wounding without the influence of general anesthesia induces an age-dependent Aβ accumulation and cognitive impairment in mice. We therefore set out to assess the effects of peripheral surgical wounding, in the absence of general anesthesia, on neuroinflammation in mice with different ages. Abdominal surgery under local anesthesia was established in 9 and 18 month-old mice. The levels of tumor necrosis factor-α (TNF-α), interleukin-6 (IL-6), Iba1 positive cells (the marker of microglia activation), CD33, and cognitive function in mice were determined. The peripheral surgical wounding increased the levels of TNF-α, IL-6, and Iba1 positive cells in the hippocampus of both 9 and 18 month-old mice, and age potentiated these effects. The peripheral surgical wounding increased the levels of CD33 in the hippocampus of 18, but not 9, month-old mice. Finally, anti-inflammatory drug ibuprofen ameliorated the peripheral surgical wounding-induced cognitive impairment in 18 month-old mice. These data suggested that the peripheral surgical wounding could induce an age-dependent neuroinflammation and elevation of CD33 levels in the hippocampus of mice, which could lead to cognitive impairment in aged mice. Pending further studies, anti-inflammatory therapies may reduce the risk of postoperative cognitive dysfunction in elderly patients.

## Introduction

Post-operative cognitive dysfunction (POCD) is one of the most common post-operative complications in senior patients [1], and is associated with increased cost, morbidity, and mortality [2], [3], [4]. However, the neuropathogenesis of POCD remains largely to be determined and this gap in knowledge impedes the further research of POCD.

In our previous studies, we have established a pre-clinical model of open abdominal surgery under local anesthesia in mice to determine the effects of peripheral surgical wounding without the influence of general anesthesia on Aβ accumulation and cognitive impairment [5]. We have found that the peripheral surgical wounding without the influence of general anesthesia can induce an age dependent Aβ accumulation and cognitive impairment [5].

Neuroinflammation, including increases in levels of pro-inflammatory cytokine tumor necrosis factor-α (TNF-α) and interleukin-6 (IL-6), as well as microglia activation, has been reported to contribute to the cognitive impairment induced by surgery under anesthesia ([6], [7], [8], [9], reviewed in [10]). However, whether the peripheral surgical wounding without the influence of general anesthesia can induce an age dependent neuroinflammation has not been investigated.

Therefore, we studied the neuropathogenesis of POCD by assessing whether the peripheral surgical wounding without the influence of general anesthesia could induce an age-dependent neuroinflammation in mice. Finally, ibuprofen is a non-steroidal anti-inflammatory drug (NSAIDs). We therefore assessed whether ibuprofen could attenuate the peripheral surgical wounding-induced cognitive impairment in the 18 month-old mice.

We chose TNF-α and IL-6 in the current studies because these two pre-inflammatory cytokines have been shown to be associated with the neuroinflammation associated with surgery under anesthesia ([6], [7], [8], [9], [11], reviewed in [10]). Finally, *CD33* is a newly suggested AD associated gene [12]. Protein CD33 is a member of the SIGLEC family of lectins can bind to sialic acid and regulate the innate immune system [13]. Moreover, a recent study has shown that the reduction in the protein levels of *CD33* can ameliorate Aβ pathology [14]. Both Aβ accumulation (reviewed in [15]) and neuroinflammation (reviewed in [10]) have been shown to be associated with cognitive impairment. Taken together, we assessed the effects of the peripheral surgical wounding, in the absence of general anesthesia, on the levels of TNF-α, IL-6, microglia activation, and CD33 in the hippocampus of mice, as well as the cognitive function in the mice.

## Results

### The Peripheral Surgical Wounding Increased TNF-α Levels in the Hippocampus of Mice at Three and Six Hours after the Procedure

**Figure 1:**
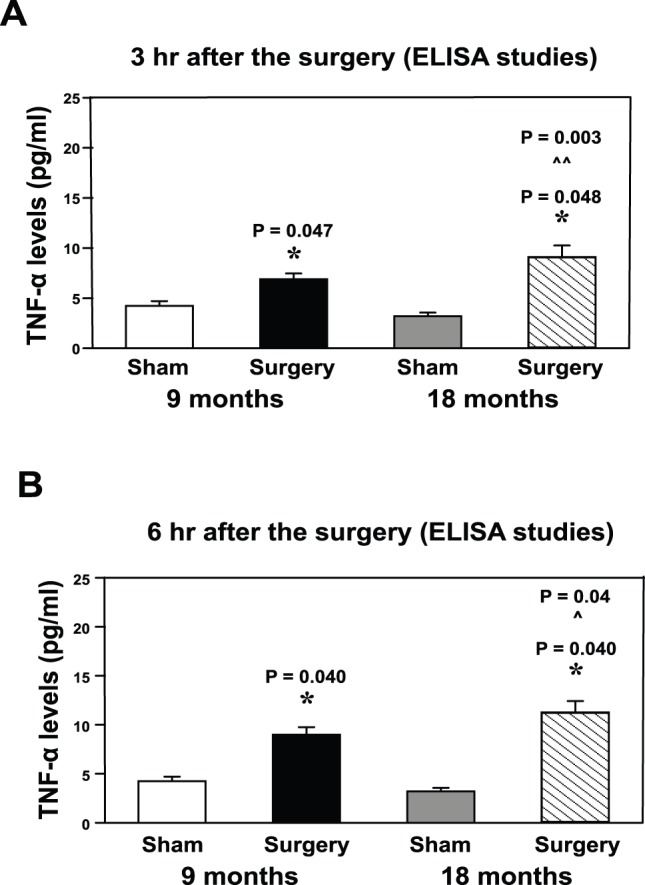
Peripheral surgical wounding increases TNF-α levels in the mouse hippocampus. **A.** Peripheral surgical wounding increases the levels of TNF-α in the hippocampus of both 9 and 18 month-old mice at three hours after the peripheral surgical wounding. Age potentiates the peripheral surgical wounding-induced elevation of TNF-α levels. **B.** Peripheral surgical wounding increases the levels of TNF-α in the hippocampus of both 9 and 18 month-old mice at six hours after the peripheral surgical wounding. Age potentiates the peripheral surgical wounding-induced elevation of TNF-α levels. (*: the difference between sham and peripheral surgical wounding; ∧ or ∧∧: the interaction between the group and the treatment). TNF-α, tumor necrosis factor-α. N = 6.

Neuroinflammation may be associated with cognitive impairment in animals [6], [8] and in patients [16]. Therefore, we assessed the effects of the peripheral surgical wounding without the influence of general anesthesia on neuroinflammation. ELISA of TNF-α showed that the peripheral surgical wounding increased the TNF-α levels in the hippocampus of both 9 (6.9 pg/ml±1.07 versus 4.2 pg/ml±0.57, P = 0.047) and 18 (9.0 pg/ml±2.51 versus 3.1 pg/ml±0.6, P = 0.048) month-old mice at three hours after the peripheral surgical wounding (Figure 1A). Two-way ANOVA indicated that age potentiated the peripheral surgical wounding-induced increases in TNF-α levels (Figure 1A, P = 0.003). The peripheral surgical wounding also increased the TNF-α levels in the hippocampus of both 9 (8.9 pg/ml±1.53 versus 4.2 pg/ml±0.57, P = 0.040) and 18 (11.1 pg/ml±2.50 versus 3.1 pg/ml±0.6, P = 0.040) month-old mice at six hours after the peripheral surgical wounding (Figure 1B). Two-way ANOVA indicated that age potentiated the peripheral surgical wounding-induced increases in TNF-α levels (Figure 1B, P = 0.04). The peripheral surgical wounding did not significantly alter the TNF-α levels in the hippocampus of mice of 9 and 18 month-old mice at 12 hours after the peripheral surgical wounding (data not shown). These data suggested that the peripheral surgical wounding without the influence of general anesthesia induced a time- and age-dependent increase in the pro-inflammatory cytokine TNF-α levels in the hippocampus of mice.

### The Peripheral Surgical Wounding Increased IL-6 Levels in the Hippocampus of Mice at 12 Hours after the Procedure

**Figure 2:**
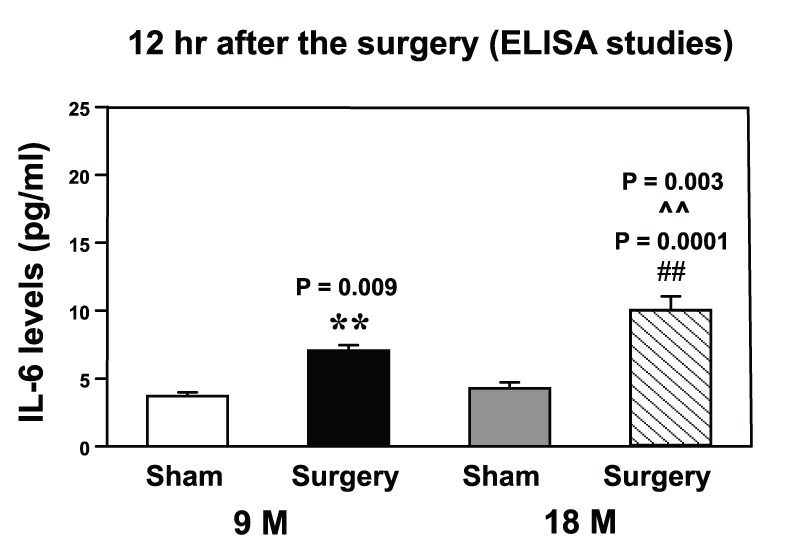
Peripheral surgical wounding increases IL-6 levels in the mouse hippocampus. Peripheral surgical wounding increases the levels of IL-6 in the hippocampus of both 9 and 18 month-old mice at 12 hours after the peripheral surgical wounding. Age potentiates the peripheral surgical wounding-induced elevation of TNF-α levels. (** or ##: the difference between sham and peripheral surgical wounding; ∧∧: the interaction between the group and the treatment). IL, interleukin. N = 6.

ELISA studies showed that the levels of pro-inflammatory cytokine IL-6 increased at 12 hours post-surgery in the hippocampus of both 9 month-old mice: 7.1 pg/ml±0.40 versus 3.1 pg/ml±0.26, **P = 0.009; and 18 month-old mice: 9.9 pg/ml±1.06 versus 4.3 pg/ml±0.44, **P = 0.0001 (Figure 2). Two-way ANOVA indicated that age potentiated the peripheral surgical wounding-induced increases in the levels of IL-6 (Figure 2, ∧∧P = 0.003) in the mouse hippocampus. The peripheral surgical wounding did not increase the IL-6 levels at three or six hours after the procedure (data not shown). These data suggested that the peripheral surgical wounding induced a time- and age-dependent increase in the pro-inflammatory cytokine IL-6 levels in the hippocampus of mice.

### The Peripheral Surgical Wounding Increased Iba1 Positive Cells in the Hippocampus of Mice at 12 Hours after the Procedure

**Figure 3:**
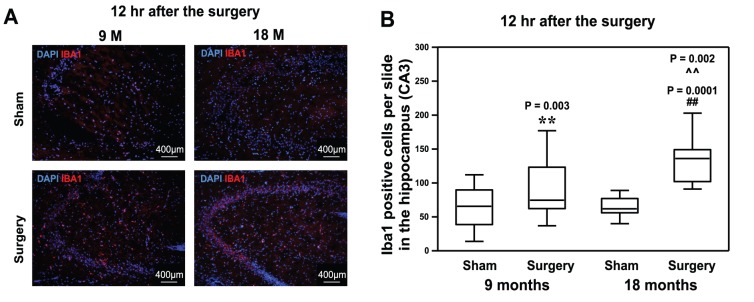
Peripheral surgical wounding increases the levels of Iba1 positive cells in the mouse hippocampus. **A.** The immunohistochemistry staining showed that peripheral surgical wounding increases the levels of Iba1 positive cells in the hippocampus of both 9 and 18 month-old mice at 12 hours after the peripheral surgical wounding as compared to sham. There are more Iba1 positive cells in the hippocampus of 18 month-old mice than that of 9 month-old mice following the peripheral surgical wounding. **B.** The quantification of the images shows that the peripheral surgical wounding increases the number of Ibal positive cells in the hippocampus of both 9 and 18 month-old mice at 12 hours after the peripheral surgical wounding as compared to sham. Age potentiates the peripheral surgical wounding-induced increases in the levels of Iba1 cells. (** or ##: the difference between sham and peripheral surgical wounding group; ∧∧: the interaction between the group and the treatment). Iba1, ionized calcium-binding adapter molecule 1. N = 6.

Immunostaining of Iba1, a marker of microglia cell activation [17], illustrated that the peripheral surgical wounding increased the number of Iba1 positive cells in the hippocampus of both 9 month-old mice: 75, 62.3–123.3 (median, interquartile range) versus 66, 38.8–89.8 (median, interquartile range), **P = 0.003; and 18 month-old mice: 136, 102.0–149.0 (median, interquartile range) versus 62, 56.0–77.0 (median, interquartile range), ##P = 0.0001 at 12 hours post-surgery (Figure 3A and 3B). Two-way ANOVA indicated that age potentiated the peripheral surgical wounding-induced increases in the levels of microglia cell activation, as evidenced by the increase in the number of Iba1 positive cells, in the hippocampus of mice (Figure 3B, ∧∧P = 0.002). These data suggested that the peripheral surgical wounding was able to induce microglia activation and age potentiated it.

### Peripheral Surgical Wounding Increased Protein Levels of Gene *CD33* in the Hippocampus of Aged Mice

**Figure 4:**
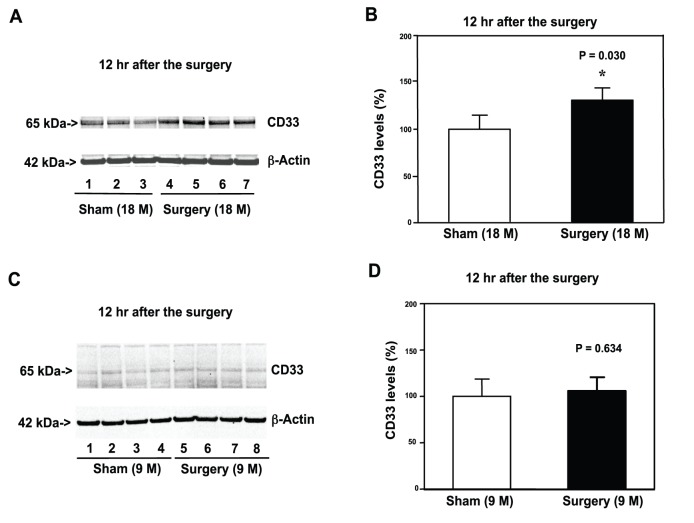
Peripheral surgical wounding only increases the levels of Iba1 positive cells in the hippocampus of 18 month-old mice. **A.** Peripheral surgical wounding increases the CD33 levels in the hippocampus of 18 month-old mice as compared to sham. There is no significant difference in β-actin levels between the peripheral surgical wounding mice and sham mice. **B.** The quantification of the Western blot shows that the peripheral surgical wounding increases the CD33 levels in the hippocampus of 18 month-old mice as compared to sham. **C.** Peripheral surgical wounding does not increase the CD33 levels in the hippocampus of 9 month-old mice as compared to sham. There is no significant difference in β-actin levels between the peripheral surgical wounding mice and sham mice. **D.** The quantification of the Western blot shows that the peripheral surgical wounding does not increase the CD33 levels in the hippocampus of 18 month-old mice as compared to sham. (*: the difference between sham and peripheral surgical wounding). N = 6.

*CD33* is a newly suggested AD associated gene [12]. As a member of the SIGLEC family of lectins, protein CD33 binds sialic acid and regulates the innate immune system [13]. We therefore assessed the effects of the peripheral surgical wounding without the influence of general anesthesia on the levels of CD33. Immunoblotting of CD33 showed that the peripheral surgical wounding increased the protein levels of *CD33* (CD33) (Figure 4A and 4B): 141%±15 versus 100%±16, *P = 0.030 in the hippocampus of 18 month-old mice at 12 hours after the procedure. However, the quantitative western blot showed that the peripheral surgical wounding did not significantly alter the CD33 levels in the hippocampus of 9 month-old mice at 12 hours after the procedure (Figure 4C and 4D). These data suggested that the peripheral surgical wounding induced an age-dependent enhancement of CD33 levels in the hippocampus of mice.

### Ibuprofen Ameliorated the Peripheral Surgical Wounding-induced Cognitive Impairment in Aged Mice

**Figure 5:**
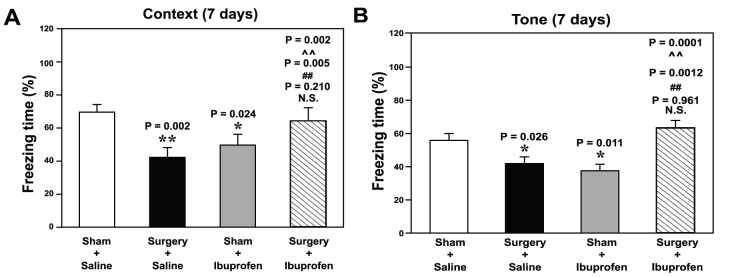
Ibuprofen attenuates the peripheral surgical wounding-induced cognitive impairment in aged mice. Peripheral surgical wounding without the influence of general anesthesia induces cognitive impairment in the context test (***A***) and tone test (***B***) of the FCS at 7 days post-surgery in 18 month-old mice. Ibuprofen attenuates the peripheral surgical wounding-induced reduction in freezing time of the context test (***A***) and the tone test (***B***) of the FCS at 7 days post-surgery (* or **: the difference between sham and peripheral surgical wounding group; ##: the difference between saline and ibuprofen treatment; ∧∧: the interaction between the group and the treatment). FCT, fear conditioning system. N = 10.

Given that the peripheral surgical wounding without the influence of general anesthesia could induce neuroinflammation (Figure 1, 2 and 3), and Aβ accumulation and cognitive impairment in aged mice [5], we assessed the cause-effect relationship by employing ibuprofen, a nonsteroidal anti-inflammatory drug (NSAID), which has been shown to attenuate neuroinflammation [18] and reduce Aβ levels [19]. We found that a treatment with ibuprofen for 7 days post-surgery ameliorated the peripheral surgical wounding-induced cognitive impairments in aged mice (Figure 5A: context test, ∧∧P = 0.002, two-way ANOVA) and (Figure 5B: tone text, ∧∧P = 0.0001, two-way ANOVA). These findings provided additional information to suggest that the peripheral surgical wounding could induce cognitive impairment in aged mice via neuroinflammation, and curbing neuroinflammation, e.g. with NSAIDs, might reduce the risk of POCD.

## Discussion

The neuropathogenesis of POCD remains largely to be determined. Neuroinflammation has been reported to be associated with POCD in humans and cognitive impairment in animals ([6], [7], [8], [9], reviewed in [10]). Our recent studies have shown that peripheral surgical wounding without the influence of general anesthesia can induce an age-dependent Aβ accumulation and cognitive impairment in mice [5]. We therefore assessed whether the peripheral surgical wounding without the influence of general anesthesia could cause an age-dependent neuroinflammation.

We found that the peripheral surgical wounding increased the levels of TNF-α (Figure 1), IL-6 (Figure 2) and Iba1 positive cells (Figure 3) in the hippocampus of both 9 and 18 month-old mice. More importantly, age potentiated the peripheral surgical wounding-induced elevations in the levels of TNF-α, IL-6 and Iba1 positive cells. These data suggested that the peripheral surgical wounding without the influence of general anesthesia could induce an age-dependent neuroinflammation (increases in pro-inflammatory cytokine levels and microglia activation), and that aged mice might be more vulnerable to the development of neuroinflammation following the insult of peripheral surgical wounding.

Note that the increases in TNF-α levels occurred at 3 and 6, but not 12 hours, after the peripheral surgical wounding, but the peripheral surgical wounding-induced increases in IL-6 levels only occurred at 12 hours after the procedure. These data suggested that the peripheral surgical wounding-induced increases in TNF-α levels could be precedent of the peripheral surgical wounding-induced increases in IL-6 levels. Terrando et al. has shown that TNF-α is upstream of IL-1 following a surgery under anesthesia in mice [6]. It is conceivable that increases in TNF-α levels following the peripheral surgical wounding could also lead to the increase in IL-6 levels. The future studies to test this hypothesis were warranted.

*CD33* is a newly suggested AD associated gene [12] and *CD33* is involved in both innate immunity and Aβ pathology [14]. A recent study has shown that the reduction in the protein levels of *CD33* (CD33) can ameliorate Aβ pathology [14]. Consistently, we found that the peripheral surgical wounding increased CD33 levels in the hippocampus of 18 month-old mice, but not in the hippocampus of the 9 month-old mice (Figure 4). These findings suggested that there was also an age-dependent changes in CD33 levels in the hippocampus of mice following the peripheral surgical wounding. Whether the difference in the CD33 levels in the hippocampus of 9 and 18 month-old mice following the peripheral surgical wounding is responsible for the difference in the neuroinflammation observed in the 9 and 18 month-old mice following the peripheral surgical wounding is not known at the present time, but warrants further investigation in the future.

Finally, non-steroidal anti-inflammatory drug ibuprofen, which has anti-inflammatory and lowers Aβ level effects [18], [19], mitigated the peripheral surgical wounding-induced cognitive impairment in the 18 month-old mice (Figure 5). These results demonstrated the cause-effect relationship of the peripheral surgical wounding-induced neuroinflammation and the peripheral surgical wounding-induced cognitive impairment. Moreover, these findings showed the potential application of anti-inflammatory treatment in preventing and treating POCD, pending further studies.

The aged TNF-α, IL-6, or CD33 knockout mice (e.g., 18 month-old mice) were not available at the time of the experiments. We therefore could not investigate the mechanism (s) underlying the effects of TNF-α, IL-6, or CD33 knockout. However, the main objective of the current studies was to determine whether there was an age-dependent change in neuroinflammation in mice following the peripheral surgical wounding without the influence of general anesthesia. We will determine whether the peripheral surgical wounding without the influence of general anesthesia can still induce cognitive impairment in aged TNF-α, IL-6, or CD33 knockout mice in our future studies.

Interestingly, there is no significant difference in the baseline levels of TNF-α, IL-6, and microglia activation (Iba1 positive cells) in the hippocampus between the 9 (adult) and 18 (aged) month-old mice in the current experiments. The exact reasons of these findings are unknown at the present time. However, it remains possible that there is still a significant difference in the baseline levels of TNF-α, IL-6, and microglia activation (Iba1 positive cells) in other brain regions of the mice, e.g., cortex, between the 9 (adult) and 18 (aged) month-old mice. The experiments to illustrate such differences may be included in future studies.

The studies have several limitations. First, we did not measure the effects of ibuprofen on the levels of TNF-α, IL-6, and microglia activation in the studies. However, the objective of the ibuprofen studies was to demonstrate the general cause-effect relationship of the peripheral surgical wounding-induced neuroinflammation and the peripheral surgical wounding-induced cognitive impairment. Second, levobupivacaine has been reported to suppress inflammation [20], , therefore, we cannot totally rule out the influence of local anesthetics on the levels of TNF-α, IL-6, and microglia activation, as well as the cognitive function observed in the current studies. However, our previous studies showed that local anesthetic bupivacaine (0.5% and 0.1 ml) did not affect the cognitive function in the mice [5]. Moreover, the sham mice also received the bupivacaine injection in the current studies. Finally, employment of EMLA might cause side effects. A case report showed that a 7-month-old girl with pulmonary dysplasia receiving inhaled nitric oxide developed cyanosis caused by methemoglobinemia after prolonged use of EMLA. We did not observe such side effects in mice after the use of EMLA in our previous experiments [5], [22].

In conclusion, we have shown that open abdominal surgery, in the absence of general anesthesia (under local anesthesia), induced an age-dependent neuroinflammation (e.g., increases in TNF-α and IL-6 levels, and microglia activation) and changes in CD33 levels in the hippocampus of mice. The aged mice are more vulnerable to the peripheral surgical wounding-induced neuroinflammation and increases in CD33 levels. Ibuprofen, an anti-inflammatory drug, ameliorated the peripheral surgical wounding-induced cognitive impairment in aged mice. Taken together, these findings carry insightful implications for the surgical care of elderly patients and suggest that the aging brain could be more susceptible to the development of neuroinflammation following peripheral surgical wounding. Pending further studies, it may be useful to consider anti-inflammatory to reduce the risk of POCD in elderly patients.

## Methods

### Mice Surgery and Treatment

All experiments followed the National Institutes of Health guidelines and the animal protocol was approved by the Massachusetts General Hospital (Boston, Massachusetts) Standing Committee on the Use of Animals in Research and Teaching. Efforts were made to minimize the number of animals used. Since it is technically difficult to perform an epidural or spinal anesthesia in mice, we have established an animal model of open abdominal surgery under local anesthesia in mice [5]. Specifically, wild type C57BL/6J female mice (9 month-old, The Jackson Laboratory, Bar Harbor, ME; and 18 month-old, National Institute of Aging, Bethesda, MD) were used in the studies. The mice were randomly assigned to a surgery or sham group by weight. The mice were gently restrained to a heating pad (37°C) using paper tape. A local anesthetic bupivacine (0.5% and 0.1 ml) was injected into the skin and subcutaneous tissue of the abdominal area. A 2.5 cm incision was made in the middle of the abdomen to open and then close the abdominal cavity in the mouse. The procedure lasted about five minutes. We did not use sedative medicine in an effort to reveal the effects of surgery alone and to minimize all other variables. EMLA cream (2.5% lidocaine and 2.5% prilocaine) was used every 8 hours for the first and second post-operative days to treat the surgery-associated pain. We did not use antibiotics because the procedure was aseptic. The non-surgery (sham) mice underwent the same procedure, only without the incision. For the interaction studies, each of the mice received ibuprofen (30 mg/kg, PO, CVS Pharmacy, Inc., Woonsocket, RI) or saline once per day for 7 days post-surgery [18], [23]. We chose abdominal surgery because it is associated with POCD in humans [24]. In addition, the surgery is easy to perform, and behavioral tests can be run without difficulty post-surgery. There was no significant difference in blood pressure, blood gas, levels of blood glucose and epinephrine, locomotor activity, or pain threshold between the surgery and sham mice, as demonstrated in our previous studies [5]. Local anesthetic induced neither neuroinflammation nor cognitive impairment in the aged mice (18 month-old mice) [5].

### Brain Tissue Lysis, Protein Amount Quantification

The harvested hippocampus tissues were homogenized on ice using immunoprecipitation buffer (10 mM Tris-HCl, pH 7.4, 150 mM NaCl, 2 mM EDTA, 0.5% Nonidet P-40) plus protease inhibitors (1 µg/ml aprotinin, 1 µg/ml leupeptin, 1 µg/ml pepstatin A). The lysates were collected, centrifuged at 12,000 rpm for 15 minutes, and quantified for total proteins by bicinchoninic acid protein assay kit (Pierce, Iselin, NJ). The hippocampus tissues were then subjected to Western blot analysis as described by Xie et al. [25].

### Western Blot Analysis

Antibody C33 (1∶200, Santa Cruz, Santa Cruz, CA, Cat. Number: sc-53199) was used to recognize protein CD33 (67 kDa). Western blot quantification was performed as described by Xie et al. [25]. Briefly, signal intensity was analyzed using a Bio-Rad (Hercules, CA) image program (Quantity One). We quantified Western blots in two steps. First, we used β-Actin levels to normalize (e.g., determining ratio of CD33 to β-Actin amount) protein levels and control for loading differences in total protein amount. Second, we presented protein level changes in the mice receiving the surgery as a percentage of those in the sham group mice. 100% of protein level changes refer to control levels for the purpose of comparison to experimental conditions.

### Immunohistochemistry

Mice were anesthetized with isoflurane briefly (1.4% isoflurane for five minutes) and perfused transcardially with heparinized saline followed by 4% paraformaldehyde in 0.1 M phosphate buffer at pH 7.4. Mouse hippocampus tissues were removed and kept at 4 degrees C in paraformaldehyde. Five µm frozen sections from mouse brain hemispheres were used for the immunohistochemistry staining. The sections were incubated with ionized calcium binding adaptor molecule 1 (Iba1) antibody (1∶500, Wako, Cat. Number: 019-19741) and secondary antibody (Alexa Fluor594, 1∶500, Invitrogen, Cat. Number: A11037). Finally, the sections were analyzed in mounting medium under a 20 × objective lens fluorescence microscope and photos of the sections were taken. An investigator who was blind to the experimental design counted the number of the Iba1 positive cells using Image J Version 1.38 (National Institutes of Health, Bethesda, MD).

### Enzyme-Linked Immunosorbent Assay (ELISA) Determination of TNF-α and IL-6

The mouse TNF-α Immunoassay kit (R&D Systems, Minneapolis, MN, Catalog number: MTA00B) and IL-6 Immunoassay kit (R&D Systems, Catalog number: M6000B) was used to determine the levels of TNF-α and IL-6 in the hippocampus tissues of the mice. Briefly, a monoclonal antibody specific for mouse TNF-α or IL-6 has been coated onto microplates. We added 50 ul of standard or samples, and then added 50 µL of assay diluent RD1-14 to the center of each well. Wells were incubated for two hours at room temperature, and washed five times. Then 100 µL of mouse TNF-α or IL-6 conjugate was added to each well and incubated for another two hours and repeated the washing. At last, wells were incubated in 100 µL of substrate solution for 30 minutes and stopped with stop solution. Determination of the optical density of each well was set at 450 nm, and corrected at 570 nm.

### Fear Conditioning System (FCS)

The FCS studies were performed as described in our previous studies with modifications [5]. Specifically, the pairing in the FCS (Stoelting Co., Wood Dale, IL) was performed at 24 hours post-surgery, mimicking the condition that patients may have difficulty learning new things after surgery. The pairing was performed twice with two minutes in between. For pairing, each mouse was allowed to explore the FCS chamber for 180 seconds before presentation of a 2-Hz pulsating tone (80 dB, 3,600 Hz) that persisted for 60 seconds. The tone was immediately followed by a mild foot shock (0.8 mA for 0.5 second). The mice were tested in FCS at 7 days after the procedure. Each mouse was allowed to stay in the same chamber for a total of 390 seconds. Cognitive functions (e.g., learning and memory) in the context and tone test were assessed by measuring the amount of time the mouse demonstrated “freezing behavior”, which is defined as a completely immobile posture except for respiratory efforts during the second 180 seconds. “Freezing behavior” was analyzed by Any-Maze (freezing on threshold: 10; freezing off threshold: 20; minimum freezing duration: one second) (Stoelting Co.). The percentage of freezing time was calculated by dividing the actual freezing time with the observed time (180 seconds).

### Statistics

The nature of the hypothesis testing was two-tailed. Data were expressed as mean ± Standard Error of the Mean (SEM). The data for the number of Iba1 positive cells were not normally distributed, thus were expressed as median and interquartile range (IQR). The number of samples varied from 6 (biochemistry studies) to 10 (behavioral studies). Two-way ANOVA was used to determine the interaction between group (9 and 18 month-old mice) and treatment (sham and peripheral surgical wounding) in FCS studies, and in the levels of TNF-α and IL-6. Student t-test was used to determine the difference in CD33 levels between the peripheral surgical wounding and sham. P values less than 0.05 (*, # and ∧) and 0.01 (**, ## and ∧∧) were considered statistically significant. Prism 6 software (La Jolla, CA, USA) software was used for all statistical analyses.
